# Bis(μ-3-hydroxy­benzoato)-κ^2^ *O* ^1^:*O* ^3^;κ^2^ *O* ^3^:*O* ^1^-bis­[bis­(1*H*-benzimidazole-κ*N* ^3^)(3-hydroxy­benzoato-κ*O*)nickel(II)] bis­(1*H*-benzimidazole-κ*N* ^3^)bis­(3-hy­droxy­benzoato-κ*O* ^1^)nickel(II) hexa­hydrate

**DOI:** 10.1107/S160053680800216X

**Published:** 2008-01-23

**Authors:** Hong Shen, Jing-Jing Nie, Jian-Rong Su, Duan-Jun Xu

**Affiliations:** a Department of Chemistry, Zhejiang University, People’s Republic of China

## Abstract

The title compound, [Ni_2_(C_7_H_5_O_3_)_4_(C_7_H_6_N_2_)_4_][Ni(C_7_H_5_O_3_)_2_(C_7_H_6_N_2_)_2_]·6H_2_O, is a mononuclear/dinuclear nickel(II) cocrystal, the two mol­ecular species inter­acting through hydrogen bonds that involve the uncoordinated water mol­ecules. In the mononuclear species, the Ni^II^ ion, located on an inversion center, is coordinated by two 1*H*-benzimidazole (bzim) ligands and two 3-hydroxy­benzoate (hba) anions in a square-planar geometry. In the centrosymmetric dinuclear species, the Ni^II^ ion is coordinated by two bzim ligands and three hba anions in a square-pyramidal geometry; of the two independent hba anions, one bridges two Ni^II^ ions with both carboxylate and hydroxyl groups whereas the other coordin­ates in a unidentate manner to the Ni^II^ ion. The apical Ni—O_hydrox­yl_ bond is 0.39 Å longer than the basal Ni—O_carbox­yl_ bonds. The face-to-face separation of 3.326 (9) Å indicates the existence of π–π stacking between parallel bzim ligands of adjacent dinuclear entities. Extensive N—H⋯O and O—H⋯O hydrogen bonds help to stabilize the crystal structure.

## Related literature

For general background, see: Deisenhofer & Michel (1989 ▶); Wu *et al.* (2003 ▶); Luo *et al.* (2004 ▶). For a related structure, see: Li *et al.* (2005 ▶).
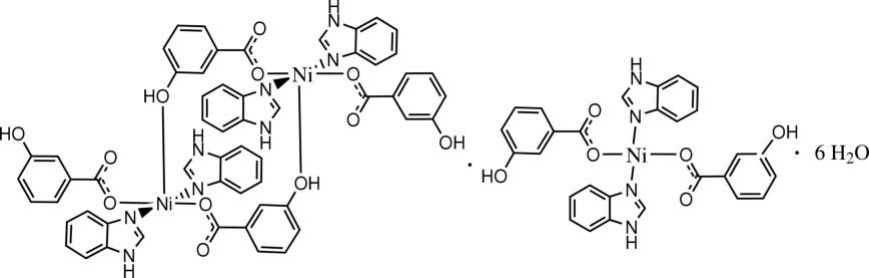


## Experimental

### 

#### Crystal data

[Ni_2_(C_7_H_5_O_3_)_4_(C_7_H_6_N_2_)_4_][Ni(C_7_H_5_O_3_)_2_(C_7_H_6_N_2_)_2_]·6H_2_O
*M* *_r_* = 1815.65
Triclinic, 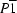

*a* = 9.9926 (12) Å
*b* = 12.9504 (15) Å
*c* = 17.069 (2) Å
α = 100.05 (2)°
β = 104.88 (3)°
γ = 101.75 (2)°
*V* = 2029.1 (6) Å^3^
*Z* = 1
Mo *K*α radiation
μ = 0.78 mm^−1^
*T* = 291 (2) K
0.36 × 0.30 × 0.22 mm

#### Data collection

Rigaku R-AXIS RAPID IP diffractometer
Absorption correction: multi-scan (*ABSCOR*; Higashi, 1995 ▶) *T*_min_ = 0.728, *T*_max_ = 0.840
17583 measured reflections
7894 independent reflections
5818 reflections with *I* > 2σ(*I*)
*R*_int_ = 0.043

#### Refinement

*R*[*F* ^2^ > 2σ(*F* ^2^)] = 0.052
*wR*(*F* ^2^) = 0.151
*S* = 1.10
7894 reflections
556 parameters
H-atom parameters constrained
Δρ_max_ = 0.77 e Å^−3^
Δρ_min_ = −0.49 e Å^−3^

### 

Data collection: *PROCESS-AUTO* (Rigaku, 1998 ▶); cell refinement: *PROCESS-AUTO*; data reduction: *CrystalStructure* (Rigaku/MSC, 2002 ▶); program(s) used to solve structure: *SIR92* (Altomare *et al.*, 1993 ▶); program(s) used to refine structure: *SHELXL97* (Sheldrick, 2008 ▶); molecular graphics: *ORTEP-3 for Windows* (Farrugia, 1997 ▶); software used to prepare material for publication: *WinGX* (Farrugia, 1999 ▶).

## Supplementary Material

Crystal structure: contains datablocks I, global. DOI: 10.1107/S160053680800216X/ng2421sup1.cif

Structure factors: contains datablocks I. DOI: 10.1107/S160053680800216X/ng2421Isup2.hkl

Additional supplementary materials:  crystallographic information; 3D view; checkCIF report

## Figures and Tables

**Table 1:** Selected bond lengths (Å)

Ni1—O1	1.914 (2)
Ni1—N13	1.994 (3)
Ni2—O4	1.960 (2)
Ni2—O7	1.961 (2)
Ni2—N23	1.980 (3)
Ni2—N33	1.983 (3)
Ni2—O9^i^	2.349 (3)

Symmetry code: (i) 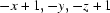.

**Table 2:** Hydrogen-bond geometry (Å, °)

*D*—H⋯*A*	*D*—H	H⋯*A*	*D*⋯*A*	*D*—H⋯*A*
N11—H11⋯O3*W*	0.86	2.07	2.906 (6)	163
N21—H21⋯O1*W*	0.86	2.02	2.867 (5)	168
N31—H31⋯O2*W*	0.86	2.02	2.866 (5)	167
O3—H3*O*⋯O8	0.86	1.75	2.609 (4)	174
O6—H6*O*⋯O2^ii^	0.99	1.80	2.783 (5)	175
O9—H9*O*⋯O5^i^	0.95	1.68	2.610 (4)	166
O1*W*—H1*A*⋯O3^iii^	0.96	2.06	2.936 (4)	152
O1*W*—H1*B*⋯O8^iv^	0.91	2.06	2.907 (4)	156
O2*W*—H2*A*⋯O5^v^	0.92	1.88	2.780 (5)	165
O3*W*—H3*B*⋯O2^ii^	0.85	2.00	2.836 (5)	168

Symmetry codes: (i) 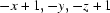; (ii) 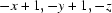; (iii) 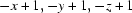; (iv) 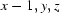; (v) 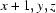.

**Fig. 1.**
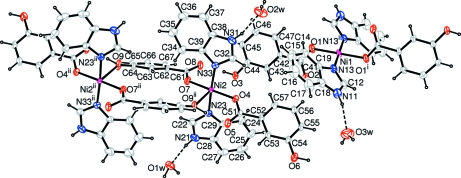
The molecular structure of the title compound with 30% probability displacement ellipsoids (arbitrary spheres for H atoms). Dashed lines indicate the hydrogen bonding [symmetry codes: (i) 2 - x,1 - y,-z; (ii) 1 - x,-y,1 - z].

**Fig. 2.**
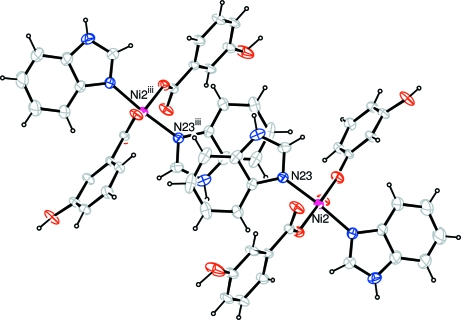
A diagram showing π-π stacking between bzim ligands [symmetry code: (iii) 1 - x,1 - y,1 - z].

## supplementary crystallographic information

### Comment

The π-π stacking is an important non-covalent interaction correlated with electron transfer in some biological systems (Deisenhofer & Michel, 1989). As part of our ongoing investigation on the nature of π-π stacking in metal complexes (Wu *et al.*, 2003; Luo *et al.*, 2004), we have recently prepared the title Ni^II^ complex with benzimidazole (bzim) ligands and determined its crystal structure.

The crystal of the title compound consists of monomeric Ni^II^ complexes, dimeric Ni^II^ complexes and lattice water molecules (Fig. 1). In the monomeric complex, the Ni^II^ ion is located on an inversion center and coordinated by two bzim ligands and two 3-hydroxybenzoate (hba) anions with a square-planar geometry (Table 1).

In the dimeric complex, each Ni^II^ ion assumes a square-pyramidal geometry formed by two bzim ligands and three hba anions, among which a pair of hba anion bridges the neighboring two Ni^II^ ions to form the centro-symmetric dimeric complex. The Ni2 ion is 0.0721 (14) Å deviated from the O4/O7/N23/N33 coordination plane towards to the O9^ii^ atom [symmetry code: (ii) 1 - *x*,-*y*,1 - *z*]. The Ni2—O9 bond in the axial direction is longer than Ni2—O4 and Ni2—O7 bonds by about 0.39 Å (Table 1), similar to that found in a complex with 2-hydroxybenzoate ligands (Li *et al.*, 2005).

The partially overlapped arrangement is observed between parallel bzim ligands of adjacent dimeric complexes (Fig. 2). The face-to-face separation of 3.326 (9) Å between N23-bzim and N23^iii^-bzim planes [symmetry code: (iii) 1 - *x*,1 - *y*,1 - *z*] indicates the existence of π-π stacking between them. The extensive N—H···O and O—H···O hydrogen bonding network helps to stabilize the crystal structure.

### Experimental

An ethanol solution (5 ml) of bzim (0.24 g, 2 mmol) was mixed with an aqueous solution (10 ml) containing sodium 3-hydroxybenzoate (0.32 g, 2 mmol) and Ni(NO_3_)_2_.6H_2_O (0.29 g, 1 mmol). The mixture was refluxed for 6 h and filtered after cooling to room temperature. The single crystals of the title compound were obtained from the filtrate after 6 d.

### Refinement

H atoms bonded to O atoms were located in a difference Fourier map and refined as riding in their as-found relative positions with *U*_iso_(H) = 1.5*U*_eq_(O). Other H atoms were placed in calculated positions with C—H = 0.93 and N—H = 0.86 Å, and refined in riding mode with *U*_iso_(H) = 1.2*U*_eq_(C,*N*).

### Crystal data

**Table:**

[Ni_2_(C_7_H_5_O_3_)_4_(C_7_H_6_N_2_)_4_][Ni(C_7_H_5_O_3_)_2_(C_7_H_6_N_2_)_2_]·6H_2_O	*Z* = 1
*M**_r_* = 1815.65	*F*_000_ = 942
Triclinic, *P*1	*D*_x_ = 1.486 Mg m^−^^3^
Hall symbol: -P 1	Mo *K*α radiation λ = 0.71073 Å
*a* = 9.9926 (12) Å	Cell parameters from 8706 reflections
*b* = 12.9504 (15) Å	θ = 3.2–25.5º
*c* = 17.069 (2) Å	µ = 0.78 mm^−^^1^
α = 100.05 (2)º	*T* = 291 (2) K
β = 104.88 (3)º	Prism, green
γ = 101.75 (2)º	0.36 × 0.30 × 0.22 mm
*V* = 2029.1 (6) Å^3^	

### Data collection

**Table:**

Rigaku R-AXIS RAPID IP diffractometer	7894 independent reflections
Radiation source: fine-focus sealed tube	5818 reflections with *I* > 2σ(*I*)
Monochromator: graphite	*R*_int_ = 0.043
Detector resolution: 10.00 pixels mm^-1^	θ_max_ = 26.0º
*T* = 291(2) K	θ_min_ = 3.1º
ω scans	*h* = −12→10
Absorption correction: multi-scan(ABSCOR; Higashi, 1995)	*k* = −15→15
*T*_min_ = 0.728, *T*_max_ = 0.840	*l* = −20→20
17583 measured reflections	

### Refinement

**Table:**

Refinement on *F*^2^	Secondary atom site location: difference Fourier map
Least-squares matrix: full	Hydrogen site location: inferred from neighbouring sites
*R*[*F*^2^ > 2σ(*F*^2^)] = 0.052	H-atom parameters constrained
*wR*(*F*^2^) = 0.151	*w* = 1/[σ^2^(*F*_o_^2^) + (0.0807*P*)^2^ + 0.2523*P*] where *P* = (*F*_o_^2^ + 2*F*_c_^2^)/3
*S* = 1.10	(Δ/σ)_max_ = 0.001
7894 reflections	Δρ_max_ = 0.77 e Å^−^^3^
556 parameters	Δρ_min_ = −0.49 e Å^−^^3^
Primary atom site location: structure-invariant direct methods	Extinction correction: none

### Special details

**Table:**

Geometry. All e.s.d.'s (except the e.s.d. in the dihedral angle between two l.s. planes) are estimated using the full covariance matrix. The cell e.s.d.'s are taken into account individually in the estimation of e.s.d.'s in distances, angles and torsion angles; correlations between e.s.d.'s in cell parameters are only used when they are defined by crystal symmetry. An approximate (isotropic) treatment of cell e.s.d.'s is used for estimating e.s.d.'s involving l.s. planes.
Refinement. Refinement of *F*^2^ against ALL reflections. The weighted *R*-factor *wR* and goodness of fit *S* are based on *F*^2^, conventional *R*-factors *R* are based on *F*, with *F* set to zero for negative *F*^2^. The threshold expression of *F*^2^ > σ(*F*^2^) is used only for calculating *R*-factors(gt) *etc*. and is not relevant to the choice of reflections for refinement. *R*-factors based on *F*^2^ are statistically about twice as large as those based on *F*, and *R*- factors based on ALL data will be even larger.

### Fractional atomic coordinates and isotropic or equivalent isotropic displacement parameters (Å^2^)

**Table:**

	*x*	*y*	*z*	*U*_iso_*/*U*_eq_	
Ni1	1.0000	0.5000	0.0000	0.03773 (18)	
Ni2	0.56466 (4)	0.17869 (3)	0.34773 (2)	0.03137 (14)	
N11	0.5837 (4)	0.3399 (3)	−0.1408 (2)	0.0653 (10)	
H11	0.5046	0.3413	−0.1750	0.078*	
N13	0.8089 (3)	0.3954 (3)	−0.05883 (17)	0.0467 (8)	
N21	0.3270 (4)	0.3388 (3)	0.4636 (2)	0.0572 (9)	
H21	0.2738	0.3397	0.4960	0.069*	
N23	0.4653 (3)	0.2811 (2)	0.39320 (17)	0.0412 (7)	
N31	0.8151 (4)	0.0406 (3)	0.2271 (2)	0.0600 (9)	
H31	0.8558	0.0370	0.1883	0.072*	
N33	0.6783 (3)	0.0884 (2)	0.30243 (18)	0.0417 (7)	
O1	1.0141 (3)	0.4464 (2)	0.09861 (15)	0.0502 (7)	
O2	0.8657 (3)	0.5328 (2)	0.14279 (15)	0.0513 (7)	
O3	0.8177 (3)	0.3899 (2)	0.39189 (16)	0.0544 (7)	
H3O	0.8298	0.3590	0.4328	0.082*	
O4	0.5044 (3)	0.2087 (2)	0.23718 (13)	0.0414 (6)	
O5	0.2715 (3)	0.1264 (2)	0.18550 (16)	0.0552 (7)	
O6	0.1196 (3)	0.3096 (3)	−0.0511 (2)	0.0861 (12)	
H6O	0.1298	0.3644	−0.0841	0.129*	
O7	0.6324 (3)	0.1567 (2)	0.46053 (14)	0.0464 (6)	
O8	0.8407 (3)	0.2847 (2)	0.50983 (16)	0.0505 (7)	
O9	0.6492 (3)	−0.0408 (2)	0.68827 (16)	0.0584 (8)	
H9O	0.6882	−0.0609	0.7382	0.088*	
O1W	0.1500 (3)	0.3747 (2)	0.5685 (2)	0.0683 (8)	
H1A	0.1355	0.4444	0.5641	0.102*	
H1B	0.0564	0.3550	0.5663	0.102*	
O2W	0.9835 (4)	0.0153 (4)	0.1156 (2)	0.0996 (12)	
H2A	1.0817	0.0434	0.1317	0.149*	
H2B	0.9734	−0.0446	0.0800	0.149*	
O3W	0.3024 (4)	0.3628 (4)	−0.2241 (3)	0.1175 (15)	
H3A	0.2405	0.3351	−0.2713	0.176*	
H3B	0.2619	0.3958	−0.1932	0.176*	
C12	0.7043 (4)	0.4195 (4)	−0.1113 (2)	0.0549 (10)	
H12	0.7140	0.4857	−0.1263	0.066*	
C14	0.8112 (5)	0.2222 (3)	−0.0099 (3)	0.0597 (11)	
H14	0.9056	0.2439	0.0246	0.072*	
C15	0.7215 (7)	0.1191 (4)	−0.0204 (3)	0.0834 (16)	
H15	0.7572	0.0709	0.0083	0.100*	
C16	0.5819 (6)	0.0870 (5)	−0.0721 (4)	0.0939 (19)	
H16	0.5267	0.0175	−0.0776	0.113*	
C17	0.5222 (6)	0.1540 (5)	−0.1155 (3)	0.0863 (17)	
H17	0.4275	0.1323	−0.1495	0.104*	
C18	0.6092 (4)	0.2549 (4)	−0.1061 (3)	0.0593 (11)	
C19	0.7499 (4)	0.2898 (3)	−0.0543 (2)	0.0510 (10)	
C22	0.3921 (4)	0.2620 (3)	0.4456 (2)	0.0506 (9)	
H22	0.3865	0.2010	0.4677	0.061*	
C24	0.5023 (4)	0.4399 (3)	0.3268 (2)	0.0511 (9)	
H24	0.5606	0.4164	0.2968	0.061*	
C25	0.4651 (6)	0.5373 (4)	0.3246 (3)	0.0720 (13)	
H25	0.5003	0.5807	0.2925	0.086*	
C26	0.3773 (6)	0.5716 (4)	0.3687 (4)	0.0809 (15)	
H26	0.3548	0.6371	0.3647	0.097*	
C27	0.3222 (5)	0.5128 (4)	0.4182 (3)	0.0701 (13)	
H27	0.2632	0.5363	0.4477	0.084*	
C28	0.3603 (4)	0.4164 (3)	0.4210 (2)	0.0493 (9)	
C29	0.4475 (4)	0.3799 (3)	0.3763 (2)	0.0407 (8)	
C32	0.7295 (4)	0.1028 (3)	0.2400 (2)	0.0457 (8)	
H32	0.7075	0.1520	0.2083	0.055*	
C34	0.7195 (7)	−0.0391 (5)	0.3972 (4)	0.103 (2)	
H34	0.6549	−0.0245	0.4256	0.124*	
C35	0.8018 (8)	−0.1100 (6)	0.4176 (6)	0.142 (3)	
H35	0.7935	−0.1427	0.4611	0.171*	
C36	0.8963 (8)	−0.1337 (6)	0.3744 (6)	0.146 (4)	
H36	0.9526	−0.1798	0.3908	0.175*	
C37	0.9083 (7)	−0.0899 (5)	0.3076 (5)	0.116 (3)	
H37	0.9683	−0.1079	0.2769	0.140*	
C38	0.8265 (5)	−0.0178 (4)	0.2884 (4)	0.0749 (15)	
C39	0.7371 (5)	0.0096 (4)	0.3329 (3)	0.0616 (12)	
C41	0.9422 (4)	0.4681 (3)	0.1481 (2)	0.0423 (8)	
C42	0.9567 (4)	0.4094 (3)	0.21743 (19)	0.0380 (8)	
C43	0.8825 (4)	0.4256 (3)	0.2744 (2)	0.0395 (8)	
H43	0.8238	0.4729	0.2703	0.047*	
C44	0.8957 (4)	0.3709 (3)	0.3382 (2)	0.0391 (8)	
C45	0.9817 (4)	0.3006 (3)	0.3451 (2)	0.0399 (8)	
H45	0.9901	0.2642	0.3879	0.048*	
C46	1.0561 (4)	0.2843 (3)	0.2872 (2)	0.0464 (9)	
H46	1.1141	0.2365	0.2913	0.056*	
C47	1.0445 (4)	0.3386 (3)	0.2237 (2)	0.0436 (8)	
H47	1.0951	0.3279	0.1855	0.052*	
C51	0.3826 (4)	0.1894 (3)	0.1838 (2)	0.0374 (8)	
C52	0.3786 (3)	0.2490 (3)	0.11543 (19)	0.0357 (7)	
C53	0.2499 (4)	0.2489 (3)	0.0608 (2)	0.0479 (9)	
H53	0.1640	0.2093	0.0645	0.058*	
C54	0.2486 (4)	0.3080 (3)	0.0001 (2)	0.0494 (10)	
C55	0.3752 (4)	0.3648 (3)	−0.0060 (2)	0.0529 (10)	
H55	0.3746	0.4029	−0.0475	0.064*	
C56	0.5029 (4)	0.3659 (3)	0.0484 (2)	0.0522 (10)	
H56	0.5886	0.4056	0.0443	0.063*	
C57	0.5053 (4)	0.3083 (3)	0.1096 (2)	0.0455 (9)	
H57	0.5923	0.3096	0.1467	0.055*	
C61	0.7452 (4)	0.2114 (3)	0.5177 (2)	0.0374 (8)	
C62	0.7627 (3)	0.1868 (3)	0.6019 (2)	0.0342 (7)	
C63	0.6919 (4)	0.0856 (3)	0.6079 (2)	0.0391 (8)	
H63	0.6324	0.0339	0.5601	0.047*	
C64	0.7111 (4)	0.0623 (3)	0.6861 (2)	0.0399 (8)	
C65	0.7907 (4)	0.1410 (3)	0.7573 (2)	0.0434 (8)	
H65	0.7984	0.1263	0.8095	0.052*	
C66	0.8591 (4)	0.2421 (3)	0.7513 (2)	0.0468 (9)	
H66	0.9121	0.2955	0.7995	0.056*	
C67	0.8492 (4)	0.2641 (3)	0.6740 (2)	0.0405 (8)	
H67	0.9003	0.3306	0.6702	0.049*	

### Atomic displacement parameters (Å^2^)

**Table:**

	*U*^11^	*U*^22^	*U*^33^	*U*^12^	*U*^13^	*U*^23^
Ni1	0.0354 (3)	0.0542 (4)	0.0231 (3)	0.0028 (3)	0.0126 (2)	0.0128 (3)
Ni2	0.0360 (2)	0.0384 (2)	0.0247 (2)	0.01420 (18)	0.00910 (17)	0.01509 (18)
N11	0.048 (2)	0.087 (3)	0.0465 (19)	−0.0023 (19)	0.0037 (16)	0.0199 (19)
N13	0.0425 (17)	0.062 (2)	0.0317 (15)	0.0035 (15)	0.0123 (13)	0.0121 (15)
N21	0.048 (2)	0.074 (2)	0.0505 (19)	0.0172 (18)	0.0222 (16)	0.0049 (18)
N23	0.0461 (17)	0.0462 (17)	0.0391 (15)	0.0189 (14)	0.0174 (13)	0.0158 (14)
N31	0.055 (2)	0.060 (2)	0.078 (2)	0.0166 (17)	0.0405 (19)	0.018 (2)
N33	0.0388 (16)	0.0455 (17)	0.0457 (16)	0.0165 (13)	0.0135 (14)	0.0159 (14)
O1	0.0484 (15)	0.0724 (18)	0.0347 (12)	0.0080 (13)	0.0205 (12)	0.0223 (13)
O2	0.0550 (16)	0.0641 (17)	0.0412 (13)	0.0153 (14)	0.0145 (12)	0.0296 (13)
O3	0.0700 (18)	0.0728 (19)	0.0500 (15)	0.0362 (15)	0.0388 (14)	0.0387 (15)
O4	0.0441 (14)	0.0541 (15)	0.0317 (11)	0.0168 (11)	0.0113 (11)	0.0206 (11)
O5	0.0471 (15)	0.0693 (18)	0.0507 (15)	0.0053 (13)	0.0101 (12)	0.0373 (14)
O6	0.0464 (17)	0.154 (3)	0.075 (2)	0.0245 (19)	0.0118 (16)	0.082 (2)
O7	0.0498 (15)	0.0560 (16)	0.0345 (12)	0.0127 (12)	0.0074 (12)	0.0226 (12)
O8	0.0555 (16)	0.0570 (16)	0.0480 (14)	0.0102 (13)	0.0212 (13)	0.0320 (13)
O9	0.0612 (17)	0.0551 (17)	0.0469 (15)	−0.0095 (13)	0.0011 (13)	0.0334 (14)
O1W	0.0643 (19)	0.0645 (19)	0.086 (2)	0.0204 (15)	0.0396 (17)	0.0140 (17)
O2W	0.057 (2)	0.135 (4)	0.092 (3)	0.016 (2)	0.023 (2)	0.000 (2)
O3W	0.086 (3)	0.174 (5)	0.090 (3)	0.054 (3)	0.011 (2)	0.027 (3)
C12	0.048 (2)	0.073 (3)	0.0360 (19)	0.003 (2)	0.0079 (17)	0.017 (2)
C14	0.069 (3)	0.057 (3)	0.056 (2)	0.013 (2)	0.027 (2)	0.015 (2)
C15	0.114 (5)	0.068 (3)	0.078 (3)	0.021 (3)	0.042 (3)	0.029 (3)
C16	0.086 (4)	0.075 (4)	0.088 (4)	−0.027 (3)	0.015 (3)	0.009 (3)
C17	0.077 (4)	0.087 (4)	0.069 (3)	−0.019 (3)	0.014 (3)	0.011 (3)
C18	0.049 (2)	0.069 (3)	0.048 (2)	−0.005 (2)	0.0139 (19)	0.006 (2)
C19	0.056 (2)	0.055 (2)	0.042 (2)	0.0028 (19)	0.0238 (18)	0.0131 (19)
C22	0.053 (2)	0.062 (3)	0.042 (2)	0.016 (2)	0.0216 (18)	0.0132 (19)
C24	0.054 (2)	0.050 (2)	0.050 (2)	0.0159 (18)	0.0106 (19)	0.0198 (19)
C25	0.079 (3)	0.057 (3)	0.079 (3)	0.024 (2)	0.006 (3)	0.031 (3)
C26	0.075 (3)	0.056 (3)	0.101 (4)	0.037 (3)	0.003 (3)	0.004 (3)
C27	0.056 (3)	0.062 (3)	0.083 (3)	0.026 (2)	0.012 (2)	−0.006 (3)
C28	0.036 (2)	0.053 (2)	0.047 (2)	0.0132 (17)	0.0039 (17)	−0.0057 (18)
C29	0.0387 (19)	0.0411 (19)	0.0385 (18)	0.0133 (15)	0.0055 (15)	0.0053 (16)
C32	0.043 (2)	0.047 (2)	0.045 (2)	0.0112 (17)	0.0123 (17)	0.0086 (17)
C34	0.127 (5)	0.109 (5)	0.155 (6)	0.087 (4)	0.099 (5)	0.093 (5)
C35	0.162 (7)	0.153 (7)	0.230 (9)	0.121 (6)	0.134 (7)	0.145 (7)
C36	0.140 (6)	0.141 (6)	0.267 (10)	0.106 (5)	0.136 (7)	0.139 (7)
C37	0.115 (5)	0.110 (5)	0.206 (8)	0.079 (4)	0.116 (5)	0.090 (5)
C38	0.063 (3)	0.067 (3)	0.125 (4)	0.034 (2)	0.054 (3)	0.044 (3)
C39	0.058 (3)	0.062 (3)	0.094 (3)	0.034 (2)	0.044 (3)	0.041 (3)
C41	0.0377 (19)	0.052 (2)	0.0285 (16)	−0.0037 (16)	0.0040 (15)	0.0133 (16)
C42	0.0417 (19)	0.0433 (19)	0.0261 (15)	0.0002 (15)	0.0109 (14)	0.0125 (15)
C43	0.045 (2)	0.046 (2)	0.0326 (17)	0.0125 (16)	0.0151 (15)	0.0185 (16)
C44	0.0402 (19)	0.047 (2)	0.0324 (17)	0.0070 (16)	0.0135 (15)	0.0163 (16)
C45	0.044 (2)	0.0407 (19)	0.0369 (17)	0.0079 (15)	0.0117 (15)	0.0177 (16)
C46	0.046 (2)	0.048 (2)	0.047 (2)	0.0162 (17)	0.0143 (17)	0.0150 (18)
C47	0.043 (2)	0.055 (2)	0.0328 (17)	0.0098 (17)	0.0157 (16)	0.0096 (17)
C51	0.042 (2)	0.044 (2)	0.0322 (16)	0.0172 (16)	0.0134 (15)	0.0152 (15)
C52	0.0392 (18)	0.0443 (19)	0.0274 (15)	0.0121 (15)	0.0122 (14)	0.0140 (15)
C53	0.043 (2)	0.069 (3)	0.0393 (19)	0.0121 (18)	0.0160 (16)	0.0290 (19)
C54	0.042 (2)	0.075 (3)	0.0387 (19)	0.0176 (19)	0.0114 (16)	0.032 (2)
C55	0.058 (2)	0.070 (3)	0.046 (2)	0.020 (2)	0.0233 (19)	0.038 (2)
C56	0.046 (2)	0.064 (3)	0.048 (2)	0.0034 (18)	0.0153 (18)	0.030 (2)
C57	0.041 (2)	0.059 (2)	0.0414 (19)	0.0148 (17)	0.0109 (16)	0.0235 (18)
C61	0.0410 (19)	0.046 (2)	0.0369 (18)	0.0182 (16)	0.0167 (16)	0.0230 (16)
C62	0.0337 (17)	0.0386 (18)	0.0365 (17)	0.0126 (14)	0.0123 (14)	0.0191 (15)
C63	0.0396 (19)	0.0419 (19)	0.0280 (16)	0.0005 (15)	0.0010 (14)	0.0141 (15)
C64	0.0353 (18)	0.048 (2)	0.0390 (18)	0.0066 (15)	0.0094 (15)	0.0242 (17)
C65	0.053 (2)	0.052 (2)	0.0296 (16)	0.0156 (18)	0.0120 (16)	0.0180 (17)
C66	0.057 (2)	0.045 (2)	0.0319 (17)	0.0113 (18)	0.0063 (17)	0.0070 (16)
C67	0.042 (2)	0.0381 (19)	0.0420 (19)	0.0092 (15)	0.0109 (16)	0.0163 (16)

### Geometric parameters (Å, °)

**Table:**

Ni1—O1^i^	1.914 (2)	C24—C29	1.382 (5)
Ni1—O1	1.914 (2)	C24—C25	1.390 (6)
Ni1—N13^i^	1.994 (3)	C24—H24	0.9300
Ni1—N13	1.994 (3)	C25—C26	1.384 (7)
Ni2—O4	1.960 (2)	C25—H25	0.9300
Ni2—O7	1.961 (2)	C26—C27	1.374 (7)
Ni2—N23	1.980 (3)	C26—H26	0.9300
Ni2—N33	1.983 (3)	C27—C28	1.382 (6)
Ni2—O9^ii^	2.349 (3)	C27—H27	0.9300
N11—C12	1.330 (5)	C28—C29	1.396 (5)
N11—C18	1.375 (6)	C32—H32	0.9300
N11—H11	0.8600	C34—C35	1.383 (7)
N13—C12	1.320 (5)	C34—C39	1.388 (6)
N13—C19	1.398 (5)	C34—H34	0.9300
N21—C22	1.329 (5)	C35—C36	1.388 (9)
N21—C28	1.376 (5)	C35—H35	0.9300
N21—H21	0.8600	C36—C37	1.379 (8)
N23—C22	1.318 (4)	C36—H36	0.9300
N23—C29	1.395 (4)	C37—C38	1.388 (7)
N31—C32	1.321 (5)	C37—H37	0.9300
N31—C38	1.390 (6)	C38—C39	1.373 (6)
N31—H31	0.8600	C41—C42	1.507 (4)
N33—C32	1.320 (4)	C42—C43	1.379 (5)
N33—C39	1.389 (5)	C42—C47	1.391 (5)
O1—C41	1.273 (4)	C43—C44	1.392 (4)
O2—C41	1.244 (4)	C43—H43	0.9300
O3—C44	1.369 (4)	C44—C45	1.373 (5)
O3—H3O	0.8583	C45—C46	1.394 (5)
O4—C51	1.265 (4)	C45—H45	0.9300
O5—C51	1.247 (4)	C46—C47	1.385 (5)
O6—C54	1.365 (4)	C46—H46	0.9300
O6—H6O	0.9838	C47—H47	0.9300
O7—C61	1.262 (4)	C51—C52	1.504 (4)
O8—C61	1.254 (4)	C52—C57	1.378 (5)
O9—C64	1.364 (4)	C52—C53	1.381 (5)
O9—H9O	0.9450	C53—C54	1.390 (4)
O1W—H1A	0.9556	C53—H53	0.9300
O1W—H1B	0.9067	C54—C55	1.367 (5)
O2W—H2A	0.9226	C55—C56	1.368 (5)
O2W—H2B	0.8684	C55—H55	0.9300
O3W—H3A	0.8443	C56—C57	1.382 (4)
O3W—H3B	0.8519	C56—H56	0.9300
C12—H12	0.9300	C57—H57	0.9300
C14—C19	1.388 (6)	C61—C62	1.501 (4)
C14—C15	1.402 (6)	C62—C63	1.391 (4)
C14—H14	0.9300	C62—C67	1.391 (5)
C15—C16	1.378 (8)	C63—C64	1.394 (4)
C15—H15	0.9300	C63—H63	0.9300
C16—C17	1.365 (8)	C64—C65	1.374 (5)
C16—H16	0.9300	C65—C66	1.382 (5)
C17—C18	1.373 (6)	C65—H65	0.9300
C17—H17	0.9300	C66—C67	1.380 (5)
C18—C19	1.388 (6)	C66—H66	0.9300
C22—H22	0.9300	C67—H67	0.9300
			
O1^i^—Ni1—O1	180.0	N23—C29—C28	108.3 (3)
O1^i^—Ni1—N13^i^	91.67 (11)	N33—C32—N31	113.5 (3)
O1—Ni1—N13^i^	88.33 (11)	N33—C32—H32	123.3
O1^i^—Ni1—N13	88.33 (11)	N31—C32—H32	123.3
O1—Ni1—N13	91.67 (11)	C35—C34—C39	117.5 (5)
N13^i^—Ni1—N13	180.0	C35—C34—H34	121.2
O4—Ni2—O7	176.81 (10)	C39—C34—H34	121.2
O4—Ni2—N23	91.29 (11)	C34—C35—C36	121.4 (6)
O7—Ni2—N23	87.74 (11)	C34—C35—H35	119.3
O4—Ni2—N33	87.26 (11)	C36—C35—H35	119.3
O7—Ni2—N33	93.42 (11)	C37—C36—C35	121.1 (5)
N23—Ni2—N33	174.55 (12)	C37—C36—H36	119.4
O4—Ni2—O9^ii^	89.67 (9)	C35—C36—H36	119.4
O7—Ni2—O9^ii^	93.33 (10)	C36—C37—C38	116.8 (5)
N23—Ni2—O9^ii^	88.21 (12)	C36—C37—H37	121.6
N33—Ni2—O9^ii^	97.04 (12)	C38—C37—H37	121.6
C12—N11—C18	107.1 (3)	C39—C38—C37	122.4 (5)
C12—N11—H11	126.4	C39—C38—N31	106.2 (4)
C18—N11—H11	126.4	C37—C38—N31	131.3 (5)
C12—N13—C19	104.6 (3)	C38—C39—C34	120.5 (4)
C12—N13—Ni1	123.2 (3)	C38—C39—N33	108.7 (4)
C19—N13—Ni1	132.1 (3)	C34—C39—N33	130.8 (4)
C22—N21—C28	107.6 (3)	O2—C41—O1	124.9 (3)
C22—N21—H21	126.2	O2—C41—C42	120.4 (3)
C28—N21—H21	126.2	O1—C41—C42	114.7 (3)
C22—N23—C29	105.4 (3)	C43—C42—C47	120.1 (3)
C22—N23—Ni2	123.5 (3)	C43—C42—C41	120.0 (3)
C29—N23—Ni2	131.0 (2)	C47—C42—C41	119.9 (3)
C32—N31—C38	106.5 (3)	C42—C43—C44	119.8 (3)
C32—N31—H31	126.8	C42—C43—H43	120.1
C38—N31—H31	126.8	C44—C43—H43	120.1
C32—N33—C39	105.0 (3)	O3—C44—C45	122.6 (3)
C32—N33—Ni2	124.6 (2)	O3—C44—C43	116.6 (3)
C39—N33—Ni2	129.9 (3)	C45—C44—C43	120.8 (3)
C41—O1—Ni1	122.7 (2)	C44—C45—C46	119.1 (3)
C44—O3—H3O	116.4	C44—C45—H45	120.4
C51—O4—Ni2	132.3 (2)	C46—C45—H45	120.4
C54—O6—H6O	112.3	C47—C46—C45	120.7 (3)
C61—O7—Ni2	127.1 (2)	C47—C46—H46	119.6
C64—O9—Ni2^ii^	146.4 (2)	C45—C46—H46	119.6
C64—O9—H9O	114.9	C46—C47—C42	119.4 (3)
Ni2^ii^—O9—H9O	92.5	C46—C47—H47	120.3
H1A—O1W—H1B	86.3	C42—C47—H47	120.3
H2A—O2W—H2B	100.9	O5—C51—O4	124.4 (3)
H3A—O3W—H3B	107.0	O5—C51—C52	120.6 (3)
N13—C12—N11	113.5 (4)	O4—C51—C52	115.1 (3)
N13—C12—H12	123.3	C57—C52—C53	119.5 (3)
N11—C12—H12	123.3	C57—C52—C51	119.3 (3)
C19—C14—C15	115.6 (5)	C53—C52—C51	121.1 (3)
C19—C14—H14	122.2	C52—C53—C54	120.1 (3)
C15—C14—H14	122.2	C52—C53—H53	120.0
C16—C15—C14	121.9 (5)	C54—C53—H53	120.0
C16—C15—H15	119.0	O6—C54—C55	121.5 (3)
C14—C15—H15	119.0	O6—C54—C53	118.8 (3)
C17—C16—C15	122.1 (5)	C55—C54—C53	119.7 (3)
C17—C16—H16	118.9	C54—C55—C56	120.4 (3)
C15—C16—H16	118.9	C54—C55—H55	119.8
C16—C17—C18	116.5 (5)	C56—C55—H55	119.8
C16—C17—H17	121.8	C55—C56—C57	120.3 (3)
C18—C17—H17	121.8	C55—C56—H56	119.9
C17—C18—N11	131.2 (4)	C57—C56—H56	119.9
C17—C18—C19	122.8 (5)	C52—C57—C56	119.9 (3)
N11—C18—C19	106.1 (3)	C52—C57—H57	120.0
C18—C19—C14	121.0 (4)	C56—C57—H57	120.0
C18—C19—N13	108.7 (4)	O8—C61—O7	125.4 (3)
C14—C19—N13	130.3 (4)	O8—C61—C62	118.3 (3)
N23—C22—N21	113.0 (3)	O7—C61—C62	116.3 (3)
N23—C22—H22	123.5	C63—C62—C67	119.7 (3)
N21—C22—H22	123.5	C63—C62—C61	119.6 (3)
C29—C24—C25	116.0 (4)	C67—C62—C61	120.7 (3)
C29—C24—H24	122.0	C62—C63—C64	119.4 (3)
C25—C24—H24	122.0	C62—C63—H63	120.3
C26—C25—C24	121.9 (5)	C64—C63—H63	120.3
C26—C25—H25	119.0	O9—C64—C65	122.3 (3)
C24—C25—H25	119.0	O9—C64—C63	117.3 (3)
C27—C26—C25	122.7 (4)	C65—C64—C63	120.4 (3)
C27—C26—H26	118.7	C64—C65—C66	119.9 (3)
C25—C26—H26	118.7	C64—C65—H65	120.0
C26—C27—C28	115.4 (4)	C66—C65—H65	120.0
C26—C27—H27	122.3	C67—C66—C65	120.3 (3)
C28—C27—H27	122.3	C67—C66—H66	119.8
N21—C28—C27	131.4 (4)	C65—C66—H66	119.8
N21—C28—C29	105.8 (3)	C66—C67—C62	120.0 (3)
C27—C28—C29	122.8 (4)	C66—C67—H67	120.0
C24—C29—N23	130.5 (3)	C62—C67—H67	120.0
C24—C29—C28	121.2 (3)		

Symmetry codes: (i) −*x*+2, −*y*+1, −*z*; (ii) −*x*+1, −*y*, −*z*+1.

### Hydrogen-bond geometry (Å, °)

**Table:**

*D*—H···*A*	*D*—H	H···*A*	*D*···*A*	*D*—H···*A*
N11—H11···O3W	0.86	2.07	2.906 (6)	163
N21—H21···O1W	0.86	2.02	2.867 (5)	168
N31—H31···O2W	0.86	2.02	2.866 (5)	167
O3—H3O···O8	0.86	1.75	2.609 (4)	174
O6—H6O···O2^iii^	0.99	1.80	2.783 (5)	175
O9—H9O···O5^ii^	0.95	1.68	2.610 (4)	166
O1W—H1A···O3^iv^	0.96	2.06	2.936 (4)	152
O1W—H1B···O8^v^	0.91	2.06	2.907 (4)	156
O2W—H2A···O5^vi^	0.92	1.88	2.780 (5)	165
O3W—H3B···O2^iii^	0.85	2.00	2.836 (5)	168

Symmetry codes: (iii) −*x*+1, −*y*+1, −*z*; (ii) −*x*+1, −*y*, −*z*+1; (iv) −*x*+1, −*y*+1, −*z*+1; (v) *x*−1, *y*, *z*; (vi) *x*+1, *y*, *z*.
